# Cyclovirobuxine D suppresses cancer stemness in osteosarcoma with implication of the noncanonical NF-kappaB pathway

**DOI:** 10.3389/fphar.2026.1746984

**Published:** 2026-03-06

**Authors:** Jinku Guo, Yingfeng Cao, Kaipen Jin, Bing Liu, Wei Wang, Chen Chen, Jun Xie, Ankai Xu

**Affiliations:** 1 Department of Orthopedics, The Quzhou Affiliated Hospital of Wenzhou Medical University, Quzhou People’s Hospital, Quzhou, Zhejiang, China; 2 Department of Anesthesia, Shulan (Hangzhou) Hospital, Shulan International Medical College, Zhejiang Shuren University, Hangzhou, China; 3 Department of Orthopedic Surgery, The Second Affiliated Hospital, Zhejiang University School of Medicine, Hangzhou, Zhejiang, China; 4 Orthopedics Research Institute of Zhejiang University, Hangzhou, Zhejiang, China; 5 Key Laboratory of Motor System Disease Research and Precision Therapy of Zhejiang Province, Hangzhou, Zhejiang, China; 6 Clinical Research Center of Motor System Disease of Zhejiang Province, Hangzhou, China; 7 Department of Nursing, The Quzhou Affiliated Hospital of Wenzhou Medical University, Quzhou People’s Hospital, Quzhou, Zhejiang, China

**Keywords:** ALDH1A1, cyclovirobuxine D, NF-KappaB, osteosarcoma, stemness

## Abstract

**Background:**

Cyclovirobuxine D (CVB-D), an alkaloid from Buxus microphylla, exhibits anticancer effects in various tumors, but its role in osteosarcoma remains unexplored. This study investigates its efficacy and mechanism in osteosarcoma.

**Methods:**

We screened a drug library and evaluated CVB-D’s effects on osteosarcoma cell lines using CCK-8 and colony formation assays. Saos2 and K7M2 cells were selected for further analysis. Flow cytometry assessed apoptosis and cell cycle. RNA-seq identified downstream pathways, including NF-kappaB, and stemness markers (CD24, ALDH1A1). Stemness was examined via serum-free suspension culture, and NF-kappaB pathway activator Diprovocim was used in rescue experiments. A xenograft mouse model validated the findings.

**Results:**

CVB-D suppressed proliferation, stemness, and induced apoptosis in osteosarcoma. These effects may be partially mediated through the p-NF-kappaB2/NF-kappaB2 axis and were reversible upon NF-kappaB pathway activation.

**Conclusion:**

CVB-D inhibits osteosarcoma possibly via the non-canonical NF-kappaB pathway, suggesting a potential therapeutic strategy.

## 1 Introduction

Osteosarcoma is a rare bone tumor that mainly affects children, adolescents, and young adults. It grows quickly and often spreads to other parts of the body. Treatment usually involves surgery, chemotherapy, and radiation, and the prognosis varies. Despite advances in treatment, osteosarcoma remains a challenging disease with a high mortality rate (Beird et al., 2022).

Natural plant compounds have gained attention in cancer research due to their anti-cancer properties, including anti-proliferative, anti-metastatic, and apoptotic effects. They have been extensively studied for their potential in various types of cancers and have shown minimal side effects. Cyclovirobuxine D (CVB-D; Chemical Abstracts Service number: 860-79–7; C26H46N2O; molecular weight: 402.66) is a natural compound with anti-inflammatory, anti-arrhythmic, and anti-cancer properties (Guo et al., 2015; Zhang et al., 2021; Jiang et al., 2020; Zeng et al., 2021). It has potential therapeutic applications in cardiovascular diseases and cancer. In cancer research, it has shown anti-proliferative, anti-metastatic, and pro-apoptotic effects on various cancer cell lines (Zhang et al., 2021; Jiang et al., 2020; Zeng et al., 2021). However, to the best of our knowledge, there are no detailed reports on the effects of CVB-D in osteosarcoma to date.

Therefore, this study aims to evaluate the anti-tumor efficacy of CVB-D against osteosarcoma and to elucidate its potential mechanisms of action, with a particular focus on its impact on cancer stemness-related properties.

## 2 Materials and methods

### 2.1 Drugs and antibodies

The information of Stem Cell Signaling Compound Library (HY-L017) and Traditional Chinese Medicine Active Compound Library (HY-L065) were provided by MedChemExpress. After obtaining the list of intersecting Chinese herbal monomers, we conducted a literature search and identified a total of 120 related compounds that have been studied less or not at all in osteosarcoma. Subsequently, we custom-ordered a library of these 120 compounds from MedChemExpress for the next phase of experimental screening. Aladdin Industrial Corporation provided CVB-D (Catalog No. C117989) for the further research. The NF-kappaB signaling pathway activator Diprovocim (HY-123942) was purchased from MedChemExpress (Wang et al., 2018; Morin et al., 2018). Purchased primary antibodies are as follows: Phospho-NF-kappaB2 (AP1391, ABclonal, China), NF-kappaB2 (A19605, ABclonal, China), ALDH1A1 (T55141, Abmart, China), CD24 (PU940443, Abmart, China), GAPDH (AB0037, Abways, China), Cleaved-PARP (CY5035, Abways, China), Caspase 3 (CY5748, Abways, China), Cleaved- Caspase 3 (CY5501, Abways, China). Horseradish peroxidase (HRP)-conjugated secondary antibodies were obtained from Biosharp (China).

### 2.2 Cell lines and treatment

The human osteosarcoma cell lines 143B, Saos-2, the mouse osteosarcoma cell line K7M2 and human umbilical vein endothelial cell HUVEC, were obtained from Cell Bank of Shanghai Institute of Biochemistry and Cell Biology, Chinese Academy of Sciences (Shanghai, China). In general circumstances, these cells were cultured in Dulbecco’s modified eagle’s medium (DMEM) supplemented with 10% fetal bovine serum (FBS, Procell, China) and 1% penicillin-streptomycin (P/S) and maintained in a humidified atmosphere at 37 °C with 5% CO2.

### 2.3 Cell viability and proliferation

To assess the inhibitory effects of CVB-D on osteosarcoma cells, the Cell Counting Kit-8 (CCK8, APExBIO, USA) assay was used. Different concentrations of CVB-D were applied to osteosarcoma cells following the described settings, with various time intervals in 96-well plates. During the testing, after calculating the required amount of CCK8 and accounting for extra, the CCK8 reagent was diluted 10 times using complete culture medium. Then, the original culture medium was aspirated and replaced with 100 μL of diluted CCK8 working solution per well. A few wells containing PBS were also aspirated to serve as blanks, and the CCK8 working solution was used as a control. The plate was incubated at 37 °C for 2 h, and absorbance at 450 nm was measured using a microplate reader. Each value was the average of at least five replicate wells. Cell viability was calculated using the formula: (Experimental group value - Blank group value)/(Control group value - Blank group value).

For the clonogenic assay, the inhibition of cell proliferation was observed. An initial seeding of about 2000 cells per well was performed in 6-well plates, and the cells were cultured for approximately 1 week with medium changes every other day. Then, cells were fixed with a fixing solution for 1 h, gently rinsed with PBS, and then gently agitated. PBS was removed by gentle aspiration, and the cells were washed twice. Crystal violet solution (Beyotime Biotechnology, China) was added for staining for 5–10 min. After two more PBS washes, the cells were allowed to dry, photographed, and then transformed using ImageJ (version 1.53s) software. To ensure blinded assessment, compound administration was handled by a researcher aware of group allocation, whereas all outcome measures were independently conducted by a second, blinded evaluator. Colonies were quantified semi-automatically using ImageJ software. Each treatment condition was tested in three independent biological replicates.

### 2.4 Cell apoptosis detection

The Annexin V-FITC/PI Apoptosis Detection Kit (Multisciences (Lianke) Biotech, China) was utilized to assess cell apoptosis. Osteosarcoma cells were treated with CVB-D for 48 h. A flow cytometer as well as FlowJo software (Version X.0.7) were used for data collection and analysis. Three independent biological experiments were conducted. The gating strategy was as follows: the main cell population was first gated on an FSC-A vs. SSC-A dot plot to exclude debris; single cells were then selected from this population on an FSC-A vs. FSC-H dot plot to exclude doublets. Finally, within the single-cell gate, Annexin V-FITC vs. PI signals were analyzed. Cells were categorized into viable (Annexin V^−^/PI^−^), early apoptotic (Annexin V^+^/PI^−^), and late apoptotic/necrotic (Annexin V^+^/PI^+^) populations based on their staining profiles. Experiments were performed in three independent replicates.

### 2.5 Cell cycle analysis

Pre-chill anhydrous ethanol in a −20 °C refrigerator for at least 2 h. For the osteosarcoma cells in the treated 6-well plate, digest and collect cells as much as possible using trypsin without EDTA. Wash cells with PBS, then resuspend in 4 °C pre-chilled Fixation buffer or pre-chilled PBS. While gently shaking, add pre-chilled anhydrous ethanol slowly to the suspension until the final ethanol concentration reaches 70%–80%, and the solution is free of visible clumps and precipitates. Subsequently, place the suspension in a −20 °C refrigerator overnight. Before processing, wash with PBS, then wash with Staining buffer (Multisciences (Lianke) Biotech, China), and discard the supernatant while avoiding light. Resuspend in PI/RNase staining buffer (Multisciences (Lianke) Biotech, China), avoiding light, and incubate for 5–10 min before analysis. Prior to analysis, use a mesh filter to remove possible cell clumps and collect cells at a low speed whenever possible. Cell cycle analysis was performed using a flow cytometer. The data were analyzed using FlowJo software (Version X.0.7). Three independent biological experiments were conducted.

### 2.6 RNA sequencing (RNA-seq) experiments

Saos2 cells in the logarithmic growth phase were selected for treatment in 10 cm culture dishes, and the number of cells in the treatment group before processing was appropriately increased. The treatment group was exposed to 24 µM CVB-D for approximately 48 h. Cells were digested with 1.5 mL TRIzol reagent (Beyotime Biotechnology, China), collected, and stored at −80 °C. This sample collection process was repeated three times.

The subsequent steps included RNA isolation, sample quality assessment, cDNA library preparation, and RNA-seq, which were performed by The Beijing Genomics Institute (BGI) Genomics. PCA analysis and differential gene construction were conducted using the BGI Genomics online platform Dr. Tom (https://biosys.bgi.com/#/report/login). Differential gene analysis was performed using the deSEQ2 method based on count data. Genes with |log2FC| ≥1 and Q-value≤0.05 were used for subsequent KEGG enrichment analysis (Kanehisa and Goto, 2000). The volcano plot code for the differential gene analysis was obtained from the FigureYa WeChat public account (FigureYa59volcano V2).

### 2.7 Tumor sphere formation

The tumor sphere formation experiment was conducted following the methodology described in other literature (Gibbs et al., 2005; Basu-Roy et al., 2013; Rainusso et al., 2011). Approximately 5000 cells were seeded per well into six-well ultra-low attachment cell culture plates (LV BioTech, China) containing sphere-forming medium. The sphere-forming medium comprised High-glucose DMEM medium (Fude Biologic Technology, China), supplemented with 100 U/mL penicillin and 100 μg/mL streptomycin (Fude Biologic Technology, China), 2 mg/mL low molecular weight heparin sodium (Aladdin Industrial Corporation, China), 20 ng/mL recombinant human EGF (AF-100-15, PEPRO TECH, USA), and 20 ng/mL recombinant human β-FGF (AF-100-18B, PEPRO TECH, USA). Cells were cultured at 37 °C in an atmosphere containing 5% CO2 to promote tumor sphere formation. After 7–10 days, images of the cells were captured under an inverted microscope at ×100 magnification. The sizes of typical tumor spheres in each group were measured by estimating their radii using ImageJ software. When conducting spheroid formation experiments using low-adhesion 6-well plates, the seeding density per well is between 5,000 and 10,000 cells. Typically, even in the control group, fewer than 10 spheroids are formed per well under these conditions. To refine the experimental assessment of spheroid formation efficiency, we used low-adhesion 10 cm culture dishes with an initial cell count of approximately 1 × 10^5 cells per dish. Due to the larger number of cells in the culture dish, we first gently swirled the medium to ensure an even distribution of the spheroids. After randomly selecting fields of view, we estimated the total number of spheroids formed in the entire culture dish.

### 2.8 Western blotting

Cells were washed with ice-cold phosphate-buffered saline (PBS) and lysed using radioimmunoprecipitation assay buffer (RIPA, Beyotime Biotechnology, China) supplemented with protease and phosphatase inhibitors (Fude Biologic Technology, China). The extracted lysates were centrifuged, and the supernatant was collected for protein concentration quantification and dissolved in 1x sample buffer (Fude Biologic Technology, China). The extracted proteins were separated by sodium dodecyl sulfate-polyacrylamide gel electrophoresis (SDS-PAGE), then transferred onto a polyvinylidene fluoride (PVDF) membrane. The membrane was blocked with 5% skim milk or bovine serum albumin (BSA). Subsequently, the membrane was incubated overnight at 4 °C with the primary antibodies: Phospho-NF-kappaB2 (1:500), NF-kappaB2 (1:500), ALDH1A1 (1:500), CD24 (1:500), GAPDH (1:1000), Cleaved-PARP (1:500), Caspase 3 (1:500), Cleaved- Caspase 3 (1:500). After washing the membrane with TBS-T, it was incubated with HRP-conjugated secondary antibodies, followed by another wash. Finally, the membrane was incubated and exposed to ECL chemiluminescence reagent (Fude Biologic Technology, China) in the dark. Semi-quantitative analysis of the Western blot data was performed using ImageJ software (National Institutes of Health, USA) and Evolution-Capt software. Statistical graphs were generated based on the analyzed data.

### 2.9 Xenograft mice model

6-8-week-old male BALB/c mice were obtained from Shanghai SLAC Laboratory Animal Co., Ltd. All experimental procedures were approved by the Animal Ethics Committee of Quzhou People’s Hospital and the Animal Ethics Committee of the Second Affiliated Hospital of Zhejiang University School of Medicine.

In brief, approximately 5 × 10^6^ K7M2 cells were subcutaneously injected into the armpit of the mice. Once the subcutaneous tumor volume reached about 20 mm^3^, the mice were randomly assigned into three groups (with n = 5 per group, using a computer-generated random number table). Mice were treated with daily intraperitoneal injections of 8 mg/kg or 16 mg/kg of CVB-D or an equivalent volume of vehicle solution. Tumor burden was measured weekly. After 14 days of treatment, the mice were euthanized, and tumor samples were harvested for immunohistochemical staining. A single-blind design was employed: compound administration was performed by a researcher aware of group allocation, while outcome assessments (e.g., tumor volume, body weight) were conducted independently by a second researcher blinded to the treatments.

### 2.10 Immunohistochemistry (IHC) staining

For IHC, in simple terms, tumor tissues fixed in formaldehyde (48 h at room temperature) and embedded in paraffin were cut into 5 µm thick sections. The sections were deparaffinized with xylene, rehydrated in a gradient series of ethanol (100%, 95%, 85%, and 75%), incubated in 3% H2O2 for 30 min, and blocked with 3% bovine serum albumin at room temperature for 1 h. Then, the sections were immunostained with p-NF-kappaB2 antibody (1:100), CD24 antibody (1:100), and ALDH1A1 antibody (1:100) overnight at 4 °C. Subsequently, they were subjected to staining using a 3,3′-diaminobenzidine substrate kit (DA1010, Beijing Solabao Technology), and counterstained with hematoxylin at room temperature for 3 min. Photographs were taken under an optical microscope (magnification, ×200). Images were processed using ImageJ software and the “EBImage” package, and subsequently, the H-score for immunohistochemistry was calculated (Hirsch et al., 2003; Pau et al., 2010).

### 2.11 Statistical analysis and software usage

Quantitative data from at least three independent experiments are described as mean ± standard deviation (SD). For two groups, data were compared using a two-tailed Student’s test. For multiple groups, data were compared using one-way analysis of variance (ANOVA) Dunn’s *post hoc* analysis or two-way ANOVA (Two-way ANOVA) Tukey’s *post hoc* analysis. Statistical analyses were performed using the R (Version 4.0.3) or GraphPad Prism 8 software. IHC images were processed using ImageJ software and the “EBImage” package. The relative expression of proteins in protein immunoblot experiments was determined using ImageJ and Evolution-Capt software. Differences were considered statistically significant when p < 0.05.

## 3 Results

### 3.1 CVB-D inhibits proliferation of osteosarcoma cells

First, we conducted a preliminary screening of drug effectiveness using a small sample drug library, including 120 traditional Chinese medicine monomers, customized by MedChemExpress (MCE) company. Based on the fundamental effects of these drugs on cell proliferation, we initially identified 23 traditional Chinese medicine monomers that could effectively inhibit the proliferation of osteosarcoma cells at a concentration of 200 μM (200 μmol/L) within 24 h (Supplementary Table S1). With the aim of reducing the impact of non-specific cytotoxicity, a compound was deemed effective only if its mean inhibition rate was greater than 80%. Subsequently, we reduced the drug concentration and extended the exposure time for further screening, ultimately selecting several drugs for further study, including the main drug CVB-D in this research.

**FIGURE 1:**
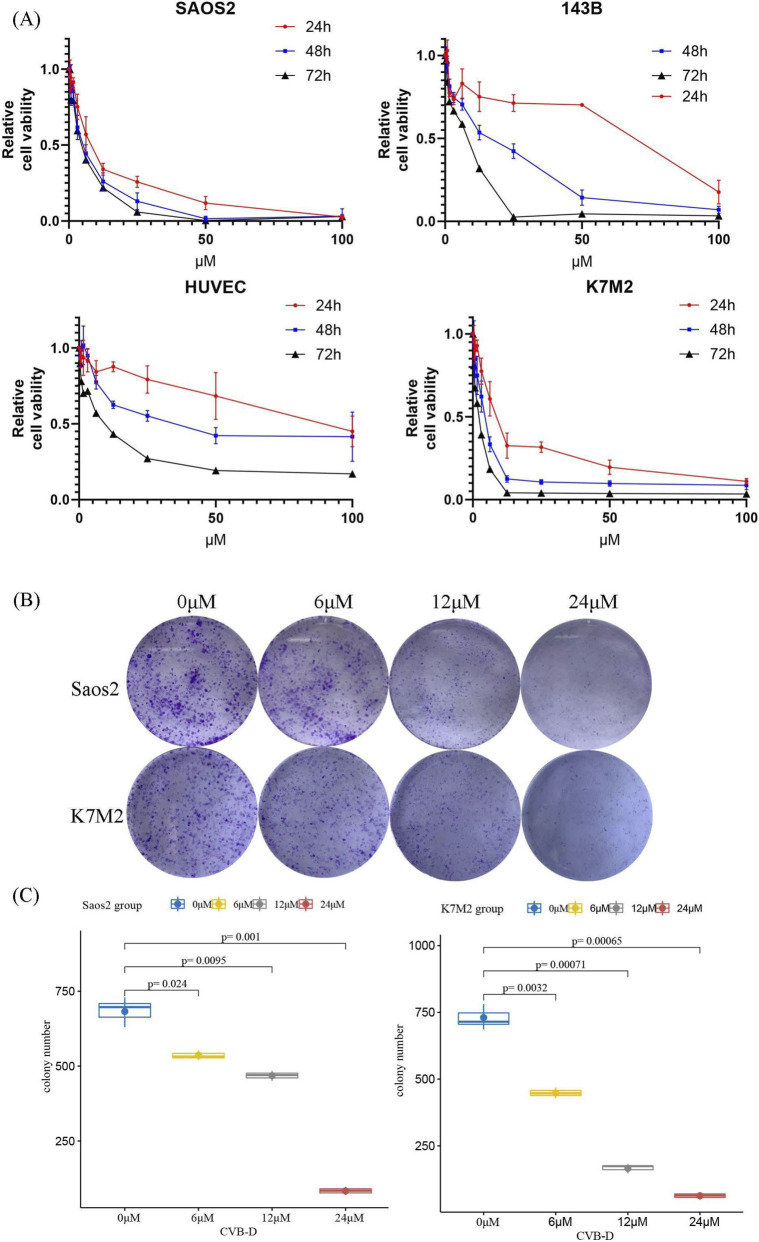
The inhibitory effect of CVB-D on cell proliferation in cell lines. **(A)** The inhibitory effect of CVB-D at different concentrations on the viability of osteosarcoma cell lines Saos2, 143B, K7M2, and control cell line HUVEC at 24h, 48h, and 72h, as determined by CCK8 assay. **(B–C)** The effect of CVB-D on the clonal formation of osteosarcoma cell lines and statistical charts. This box plots present data from three independent experiments. p < 0.05 considered statistically significant.

To further evaluate the inhibitory effect of CVB-D on cell viability, we exposed osteosarcoma cell lines Saos2, 143B, and K7M2, as well as the control cell line HUVEC, to increasing concentrations of CVB-D (0–100 µM) for 24, 48, and 72 h. The results showed that CVB-D significantly inhibited the viability of osteosarcoma cells in a dose and time-dependent manner, while its relative inhibitory effect on the non-tumor cell group cells was weaker (Figure 1A). Based on these findings, it is speculated that at its effective tumor-killing concentration, this drug induces minimal damage to blood vessels. These data lead us to cautiously infer that the drug might cause limited adverse effects on human vasculature. These observations suggest that the drug could be considered a promising candidate for clinical translation, warranting further investigation. However, it should be noted that the tumor stroma comprises a heterogeneous population of non-cancer cells. HUVECs, being derived from umbilical veins, may not fully represent the stromal cells or tissue-specific endothelial cells within the osteosarcoma microenvironment. Future studies could utilize more disease-relevant control models, such as bone-derived fibroblasts or patient-derived normal mesenchymal stem cells, for more accurate safety profiling. The IC50 of CVB-D at 48 h was 38.79 μM (95% CI: 23.73–63.40 µM) for HUVEC cells, 16.42 μM (95% CI: 8.66–31.16 µM) for 143B cells, 4.96 μM (95% CI: 4.14–5.94 µM) for Saos-2 cells, and 3.93 μM (95% CI: 0.70–21.96 µM) for K7M2 cells. Colony formation assays further confirmed the inhibitory effect of CVB-D on osteosarcoma clonal formation (Figures 1B,C).

### 3.2 CVB-D induces apoptosis in osteosarcoma cells

**FIGURE 2:**
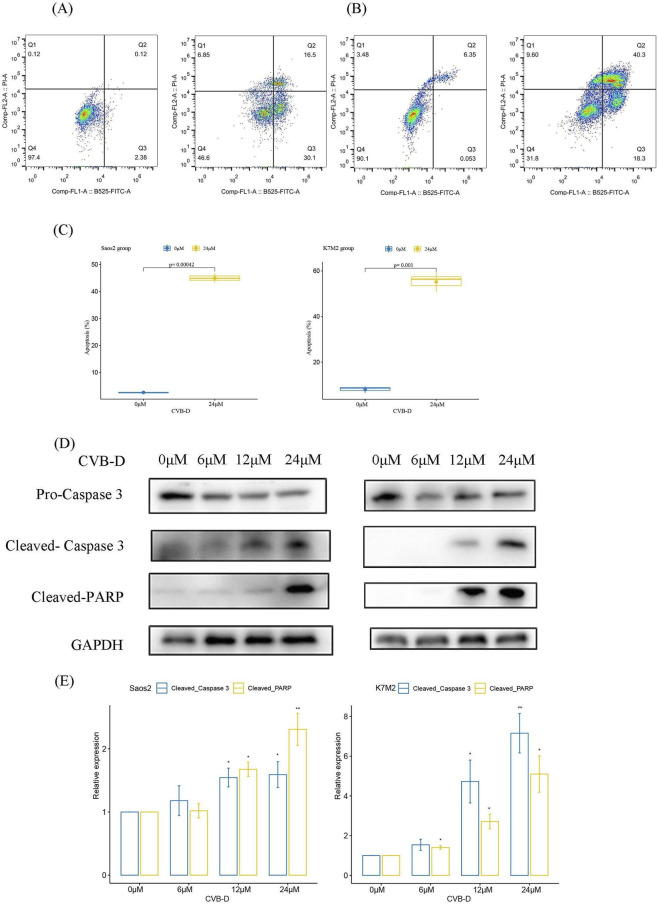
CVB-D promotes apoptosis in osteosarcoma cells. **(A)** CVB-D promotes apoptosis in Saos2 cell line. (Left: control group; Right: 24 µM CVB-D treatment for 48 h). **(B)** CVB-D promotes apoptosis in K7M2 cell line. (Left: control group; Right: 24 µM CVB-D treatment for 48 h). **(C)** Statistical chart of CVB-D-induced apoptosis in osteosarcoma cells. This box plots present data from three independent experiments. p < 0.05 considered statistically significant. **(D)** The expression levels of apoptosis-related proteins were detected by Western blotting. (Left: Saos2 cell line; Right: K7M2 cell line). **(E)** Statistical analysis of the relative expression levels of apoptosis-related proteins. The histogram contains data from three separate experiments. *P < 0.05 versus control, **P < 0.01 versus control.

Cell apoptosis is one of the key mechanisms for eliminating tumor cells. Through flow cytometry, we found that CVB-D significantly induced apoptosis in osteosarcoma cells (Figure 2). While CVB-D at 12 and 24 μM potently induced apoptosis in osteosarcoma cells, evidenced by elevated levels of cleaved-PARP and cleaved-caspase 3, the precise apoptotic signaling pathways activated have not been explored, which is a current limitation of the study (Figures 2D,E).

No consistent effect of CVB-D on cell cycle distribution was observed by flow cytometry in various osteosarcoma cell lines. (Supplementary Figure S1). CVB-D treatment exhibited differential effects on the cell cycle across osteosarcoma cell lines: it had no significant impact on K7M2 cells but altered the cell cycle distribution in Saos2 cells. The underlying mechanism for this cell line-specific disparity warrants further study.

### 3.3 Exploring the potential mechanisms of CVB-D inhibiting osteosarcoma cells through RNA-seq

**FIGURE 3:**
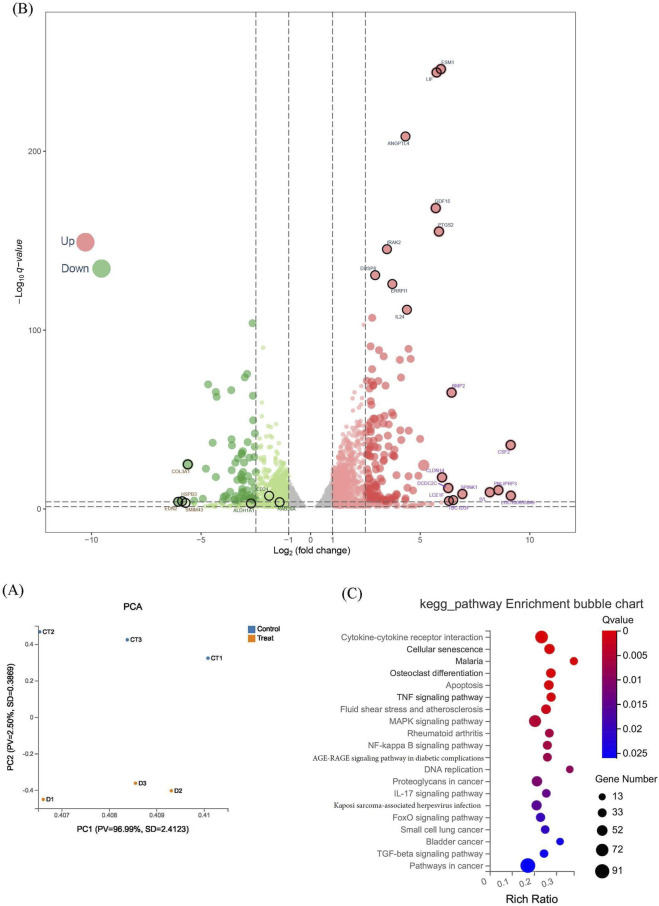
CVB-D affects osteosarcoma through RNA-seq analysis. **(A)** Volcano plot of differential genes between CVB-D-treated and control groups. Up indicates that the expression level in the drug-treated group is higher than that in the control group; Down indicates that it is lower. **(B)** PCA analysis of CVB-D-treated and control groups. **(C)** KEGG pathway enrichment analysis of differential genes in osteosarcoma cell lines under the influence of CVB-D.

We conducted next-generation sequencing of Saos2 cells treated with CVB-D (24 μM) for 48 h, along with untreated Saos2 cells, and summarized differentially expressed genes in the Supplementary Table S2. Principal component analysis (PCA) of the sequencing results is shown in Figure 3A. Additionally, the volcano plot of differential gene expression suggested that CVB-D may target ALDH1A1 and CD24, key markers of cancer stem cells, to regulate osteosarcoma stemness (Figure 3B). KEGG pathway enrichment analysis of significantly differentially expressed genes (log |FC| > 1, FDR <0.05) predicted potential mechanisms of CVB-D action (Figure 3C; Supplementary Table S3). According to the KEGG analysis, CVB-D might exert its inhibitory effect on osteosarcoma through several pathways, including but not limited to Cytokine-cytokine receptor interaction; Cellular senescence; Osteoclast differentiation; and the TNF, MAPK, NF-kappa B, AGE-RAGE, IL-17, FoxO, and TGF-beta signaling pathways.

### 3.4 CVB-D partially regulates osteosarcoma stem cells through a non-classical NF-kappaB signaling pathway

**FIGURE 4:**
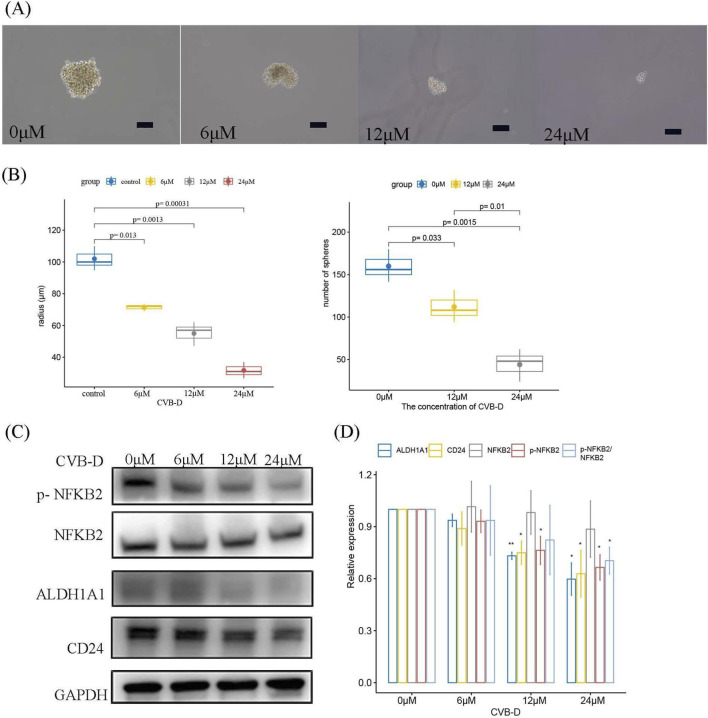
CVB-D inhibits the non-classical NF-kappaB signaling pathway and osteosarcoma stemness. **(A,B)** The effect of different concentrations of CVB-D on the tumor sphere formation of osteosarcoma cells and statistical charts, with a scale of 100 μm. This box plots present data from three independent experiments. p < 0.05 considered statistically significant. **(C)** The inhibitory effect of different concentrations of CVB-D on the non-classical NF-kappaB signaling pathway and related tumor stemness markers. **(D)** Statistical analysis of the relative expression levels of signaling pathway and related tumor stemness markers. The histogram contains data from three separate experiments. *P < 0.05 versus control, **P < 0.01 versus control.

We further observed the inhibitory effect of CVB-D on the formation of osteosarcoma tumor spheres at different concentrations. As the concentration of CVB-D increased, its inhibitory effect on the volume of tumor spheres as well as spheroid formation efficiency became more significant (Figures 4A,B). Western blot analysis confirmed that CVB-D inhibited the non-classical NF-kappaB signaling pathway (p-NF-kappaB2/NF-kappaB2 pathway) and the expression of tumor stem cell markers ALDH1A1 and CD24.

**FIGURE 5:**
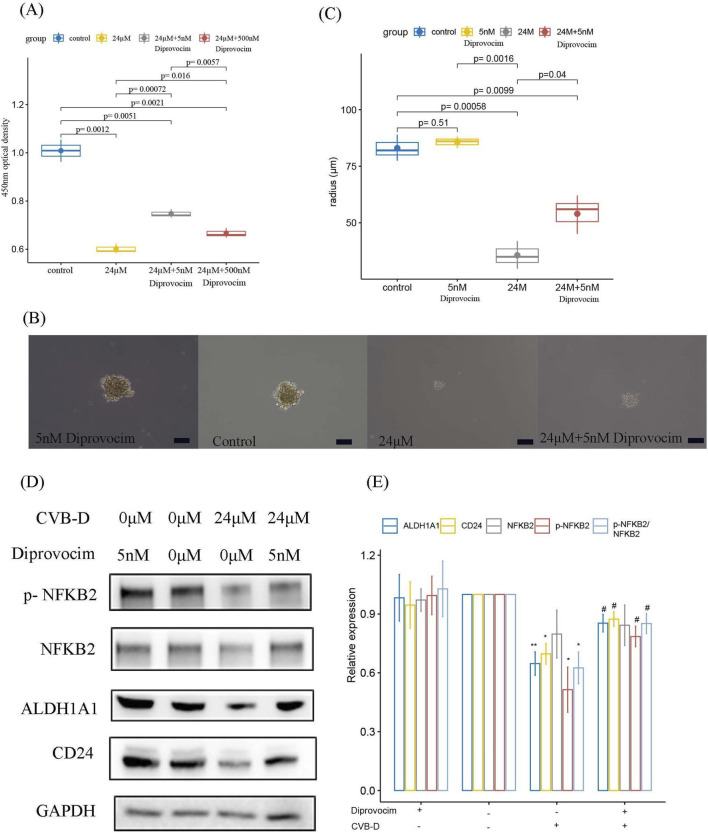
CVB-D may inhibit osteosarcoma stemness through the non-classical NF-kappaB signaling pathway. **(A)** The optimal working concentration of Diprovocim was determined using the CCK-8 assay. **(B–C)** Diprovocim restores the inhibitory effect of CVB-D on osteosarcoma sphere formation and statistical charts, with a scale of 100 μm. This box plots present data from three independent experiments. p < 0.05 considered statistically significant. **(D)** Diprovocim restores the inhibition of CVB-D on the non-classical NF-kappaB signaling pathway and osteosarcoma stemness markers. **(E)** Statistical analysis of the relative expression levels of signaling pathway and related tumor stemness markers. The histogram contains data from three separate experiments. *P < 0.05 versus control, **P < 0.01 versus control. #P < 0.05 versus treatment with CVB-D alone.

Furthermore, we conducted a rescue experiment using Diprovocim (CAS: 2170867-89-5), a TLR1/TLR2 agonist that can activate the downstream NF-kappaB signaling pathway. First, the optimal working concentration of Diprovocim was determined using the CCK-8 assay for subsequent experiments. The results showed that, compared to the low concentration (5 nM), the high concentration (500 nM) of Diprovocim exhibited a non-negligible inhibitory effect on the tumor, although Diprovocim partially counteracted the inhibitory effect of CVB-D on osteosarcoma (Figure 5A). The results showed that Diprovocim partially restored the inhibitory effect of CVB-D on tumor sphere formation (Figures 5B,C). Combined with Western blot experiments, we proposed that CVB-D may partially regulate osteosarcoma stemness through a non-classical NF-kappaB signaling pathway (Figure 5D).

### 3.5 Inhibition of heterotransplantation tumor growth by CVB-D in osteosarcoma

**FIGURE 6:**
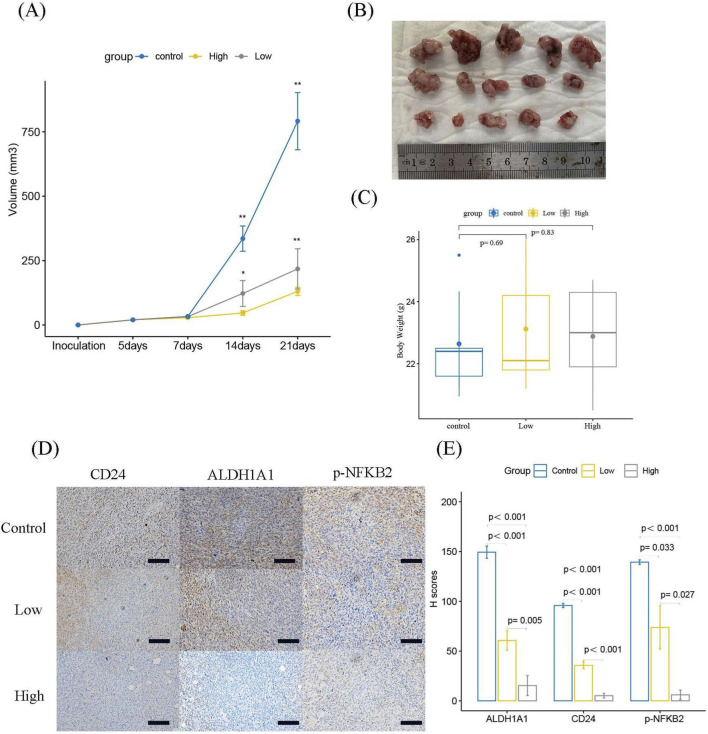
The effect of CVB-D in a xenograft mouse model. **(A)** Changes in tumor volume over time under CVB-D treatment. Drug administration was initiated on day 5 post-inoculation (designated as day 0). *p < 0.05 versus control, **p < 0.01 versus control. **(B)** Tumor sizes in mice at the time of collection. The top is the control group, the middle is the low-concentration CVB-D treatment group, and the bottom is the high-concentration CVB-D treatment group. **(C)** Statistical chart of mouse weight at adoption (p < 0.05 considered statistically significant). **(D)** Immunohistochemical images of mice, with a scale of 100 μm. **(E)** Statistical analysis of H-Scores from immunohistochemistry. p < 0.05 considered statistically significant.

We established a K7M2 heterotransplantation model in BALB/C mice to evaluate the anti-tumor ability of CVB-D. After 3 weeks, the tumor weight and size in the CVB-D treatment group were significantly smaller than those in the control group, while there was no significant difference in mouse body weight (Figures 6A–C). IHC analysis revealed that the expression levels of CD24, ALDH1A1, and p-NFKB2 were reduced in the CVB-D treatment group compared to the control group (Figures 6D,E).

## 4 Discussion

Many natural plant compounds, including traditional medicine, have gained attention in cancer research due to their anti-cancer properties. Some compounds show relatively low toxicity to normal cells. In parallel, the focus has shifted towards studying tumor stem cells as a critical factor in tumor initiation, progression, metastasis, and drug resistance (Visvader, 2011). However, there is limited research on the specific traditional medicine monomers that are effective against osteosarcoma stem cells. In this study, we used experimental methods to screen 120 traditional Chinese medicine monomers, which have been found to have inhibitory effects on tumor stem cells but have not been thoroughly studied in osteosarcoma. The results are reliable, and the negative results contribute to saving resources and time for future researchers who might otherwise attempt to use ineffective or less efficient drugs. The traditional Chinese medicine monomers identified in this study may potentially be incorporated into clinical research for novel osteosarcoma treatments, providing theoretical guidance for future clinical practice.

CVB-D, also known as cyclovirobuxine D, is the main active component of the traditional Chinese medicine, small-leaved yellow bupleurum. It is a natural compound with anti-inflammatory, anti-arrhythmic, and anti-cancer properties (Guo et al., 2015; Zhang et al., 2021; Jiang et al., 2020; Zeng et al., 2021). In particular, CVB-D has been reported to inhibit colon cancer stem cells (Jiang et al., 2020). However, there has been limited detailed research on the effects of CVB-D on osteosarcoma. In this study, we first identified the ability of CVB-D to inhibit osteosarcoma cell proliferation at relatively low concentrations through drug library screening. Subsequently, we conducted a comprehensive study of CVB-D’s inhibitory effects on osteosarcoma cell lines at different concentrations and exposure times. Furthermore, through flow cytometry, we found that CVB-D promotes apoptosis in osteosarcoma cells.

RNA-seq technology has been widely used for studying various diseases and provides an effective tool for sequencing transcripts using high-throughput sequencing technology. It enables targeted drug mechanism research. To further investigate the inhibitory mechanisms of CVB-D in osteosarcoma, we conducted RNA-seq analysis. By analyzing RNA-seq data, we obtained a landscape of transcriptome changes in osteosarcoma following CVB-D treatment, predicting potential drug targets and related oncogenic signaling pathways. We found that the osteosarcoma stem cell markers ALDH1A1 and CD24 were significantly downregulated in the CVB-D treatment group. Additionally, KEGG enrichment analysis predicted relevant signaling pathways affected by CVB-D. Through observation of tumor sphere formation effects and Western blotting experiments, we confirmed that CVB-D can inhibit osteosarcoma stemness and the non-classical NF-kappaB signaling pathway (p-NF-kappaB2/NF-kappaB2 pathway). We further conducted a recovery experiment using Diprovocim (CAS: 2170867-89–5), a TLR1/TLR2 agonist that can activate downstream NF-kappaB signaling pathways simultaneously (Wang et al., 2018; Morin et al., 2018). The results indicated that Diprovocim partially restored the inhibitory effects of CVB-D on tumor sphere formation of osteosarcoma. Finally, we performed further validation using a mouse xenograft model and immunohistochemical methods.

Gibbs and colleagues were the first to isolate osteosarcoma tumor stem cells exhibiting self-renewal and pluripotency from human samples and cell lines using the serum-free, low-attachment suspension culture method (Gibbs et al., 2005). Subsequently, in xenotransplantation assays, the ability of tumor cells to form tumor spheres under these conditions has been proven to be closely correlated with their tumorigenic potential (Rainusso et al., 2011). Further studies have since characterized the morphology and properties of these osteosarcoma tumor spheres, confirming their self-renewal capacity, enhanced drug resistance, and high expression of key embryonic stem cell marker genes (Fujii et al., 2009). Subsequently, spheroid formation under serum-free, suspension conditions has become a well-established biological property representing cellular stemness (Subramaniam et al., 2020). ALDH1A1 is a crucial member of the aldehyde dehydrogenase superfamily. It catalyzes the oxidation of retinaldehyde to retinoic acid, a key signaling molecule governing cellular differentiation and proliferation. Usually, high ALDH1A1 expression correlates with poor prognosis, tumor cell proliferation, treatment resistance, and tumorigenicity (Poturnajova et al., 2026). In osteosarcoma research, ALDH1A1 has been found to play a role in lung metastasis and chemoresistance. Furthermore, its function as an indicator of osteosarcoma stemness has been extensively reported (Honoki et al., 2010; Heim et al., 2024; Ma et al., 2019; Guo et al., 2019; Lin et al., 2022). CD24 is a cell adhesion molecule that binds to P-selectin on endothelial cells and platelets. This interaction is critical for immune cell migration and promotes cancer metastasis. In osteosarcoma, CD24^+^ cells have been identified to possess characteristics of tumor-initiating cells and are associated with resistance to drug-induced apoptosis (Zhou et al., 2020). Similarly, CD24 has also been proposed as a marker for stemness in osteosarcoma (Fujiwara et al., 2020; Yao et al., 2020). In our study, we employed ALDH1A1 and CD24 as stemness markers, utilizing the serum-free suspension spheroid formation assay to explore changes in the stemness properties of osteosarcoma. A limitation is that the current evidence would require further validation by prospectively isolating cells using flow cytometry and performing definitive *in vivo* limiting dilution assays.

This study has some limitations. First, given that Diprovocim broadly activates the NF-kappaB signaling pathway, it can only be concluded that CVB-D likely acts through the noncanonical NF-kappaB signaling pathway. Although the data suggest an association with the NF-kappaB signaling pathway, they are insufficient to establish that the effect is mediated specifically through the noncanonical pathway. Second, the RNA-seq results have not been independently validated by PCR and should therefore be interpreted as exploratory findings. Additionally, the concentration of CVB-D used in our experiments is still higher than that of some first-line osteosarcoma treatments, such as methotrexate, which we observed to have a significant inhibitory effect at the nanomolar level.

## Data Availability

The datasets presented in this study can be found in online repositories. The names of the repository/repositories and accession number(s) can be found in the article/Supplementary Material.

## Supplementary material

SUPPLEMENTARY FIGURE 1 The effect of CVB-D on the cell cycle of osteosarcoma cells. **(A)** The effect of CVB-D on the cell cycle of Saos2 cells. (Left: control group; Right: 24 µM CVB-D treatment for 48h). **(B)** The effect of CVB-D on the cell cycle of K7M2 cells. (Left: control group; Right: 24 µM CVB-D treatment for 48h). **(C)** Statistical chart of the effect of CVB-D on the cell cycle of osteosarcoma cells. The histogram contains data from three separate experiments. p < 0.05 considered statistically significant.
