# Supplementary figures and images for: Cyclovirobuxine D suppresses cancer stemness in osteosarcoma with implication of the noncanonical NF-kappaB pathway
Source: Front Pharmacol. 2026 Mar 6;17:1746984. doi: 10.3389/fphar.2026.1746984 (PMC13002614; doi:10.3389/fphar.2026.1746984)

Supplement figure 1

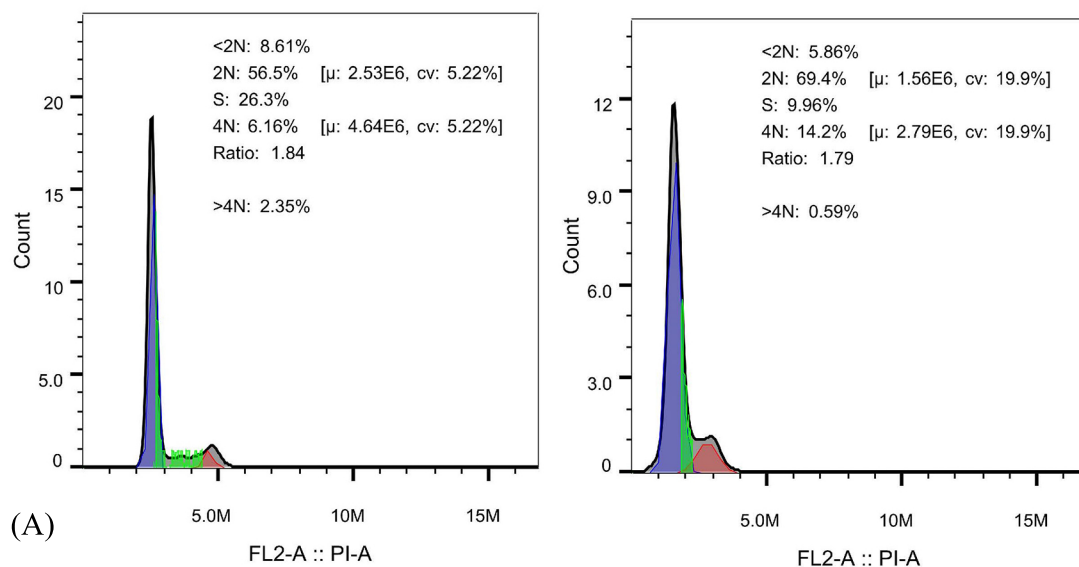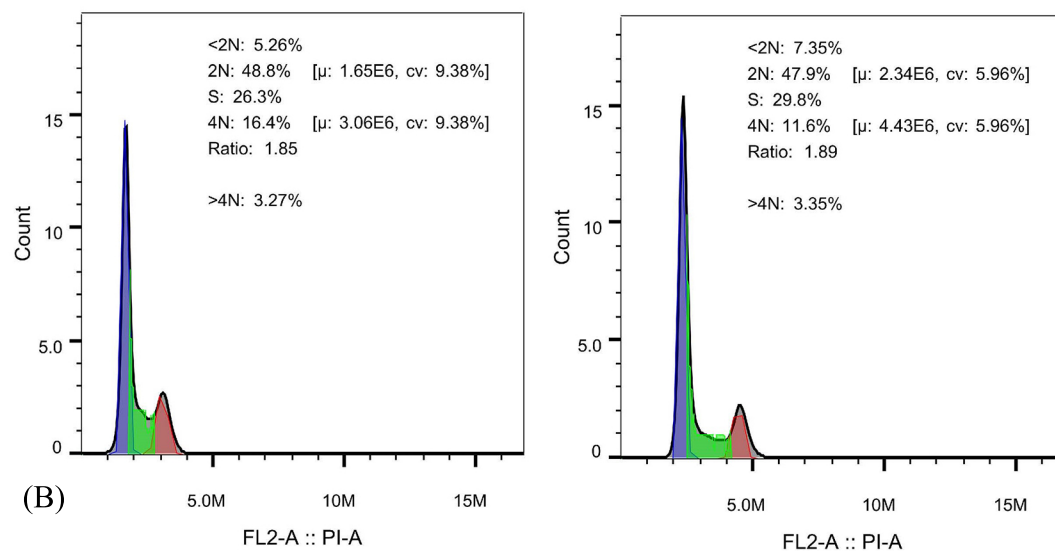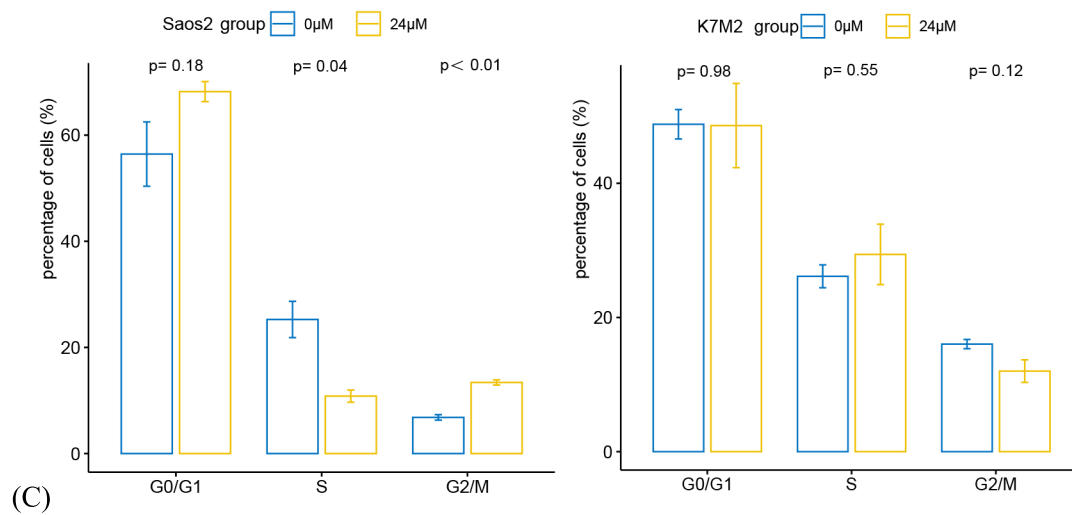

Supplement: Supplementary file 1 [file DataSheet1.pdf]
